# On the uniqueness of functional redundancy

**DOI:** 10.1038/s44185-023-00029-z

**Published:** 2023-11-08

**Authors:** Felícia M. Fischer, Francesco de Bello

**Affiliations:** 1 Centro de Investigaciones sobre Desertificación (CSIC-UV-GVA), Valencia, Spain; 2 Department of Botany, Faculty of Science, University of South Bohemia, 37005 České Budějovice, Czech Republic

**Keywords:** Community ecology, Ecosystem services, Ecology, Ecosystem ecology, Ecology

**arising from** N. Eisenhauer et al. *npj Biodiversity* https://doi.org/10.1038/s44185-023-00015-5 (2023)

Functional redundancy is increasingly being considered in ecological studies as a key component of biodiversity, potentially associated with the ability of ecosystems to buffer environmental perturbations. The recent paper by Eisenhauer et al.^1^ (hereafter “E23”), however, raises some concerns about the ecological relevance of redundancy and its potential misuses. E23^1^ suggests overall, that the concept is problematic because (1) the effects of functional redundancy are questionable, (2) the use of this term is potentially “dangerous” by implying that species are expendable, and (3) that the term “functional similarity” is preferable. While we agree that functional redundancy remains conceptually and quantitatively puzzling, we here propose clarifying various aspects of this interesting discussion to show that functional redundancy can reflect a fundamental dimension of biodiversity, beyond functional similarity.

We clearly agree with E23^1^ that the concept of functional redundancy bears a certain complexity, with multiple and sometimes contrasting interpretations. E23^1^ go one step further and questions the existence of redundancy, arguing that complete redundancy is incompatible with coexistence, citing Loreau^2^. However, a widespread view considers that redundancy is possible when species are similar in some features but dissimilar in others^3^. For instance, a similarity across species´ ecosystem effects is possible together with a dissimilarity in their environmental preferences^4^. Such redundancy can allow both coexistence and stability in ecosystem functioning^3^.

A hypothetical example (Fig. 1) can be illustrated in meadows, which are often composed of grasses and nitrogen-fixing species, with nitrogen-fixing being an “effect trait” (*sensu* Pillar et al.^4^) affecting soil fertility and plant productivity, and nitrogen fixers being a “effect group”. The loss, or decrease, of nitrogen-fixing species, for example, due to environmental perturbations, can be partially compensated by the presence of other nitrogen fixers, that are more tolerant to perturbation (Fig. 1c). This effect is generally defined as an insurance, or compensatory mechanism^3^, a key aspect for resistance and recovery after environmental perturbations. This effect would be impossible if there was only one nitrogen-fixing species (Fig. 1a) or if there were several nitrogen fixers with exactly the same response, or sensitivity, to perturbations (Fig. 1b). Clearly the presence of multiple species within each effect group increases the likelihood that they do not share the same sensitivity to perturbations. Although communities “(a)” and “(b)” would have, prior to perturbation, the same functional dissimilarity in terms of ecosystem effects (expressed, for example, through common indices for functional diversity like Rao or Functional dispersion^5^), clearly, community “(b)” has a higher likelihood of having more stable ecosystem functioning due to having more species per effect group. As such, similarity alone is not a sufficient indicator of stability, and both similarity and species diversity need to be accounted for. These two indices together are used to estimate redundancy^6,7^. Moreover, the potential for insurance mechanisms should increase when species have similar effect traits but different sensitivity to perturbations (i.e., different response traits, defined as traits responsible for adaptations to abiotic and biotic conditions, for example root depth underlying the ability to use different water resources during drought). In this sense, an important component of functional redundancy is, paradoxically, the “uniqueness” of species^8^ (Fig. 1c) being a prerequisite for its ecological effects. Ecosystem stability should, thus, be maximized when species similar in some traits are dissimilar in others.

**Fig. 1:**
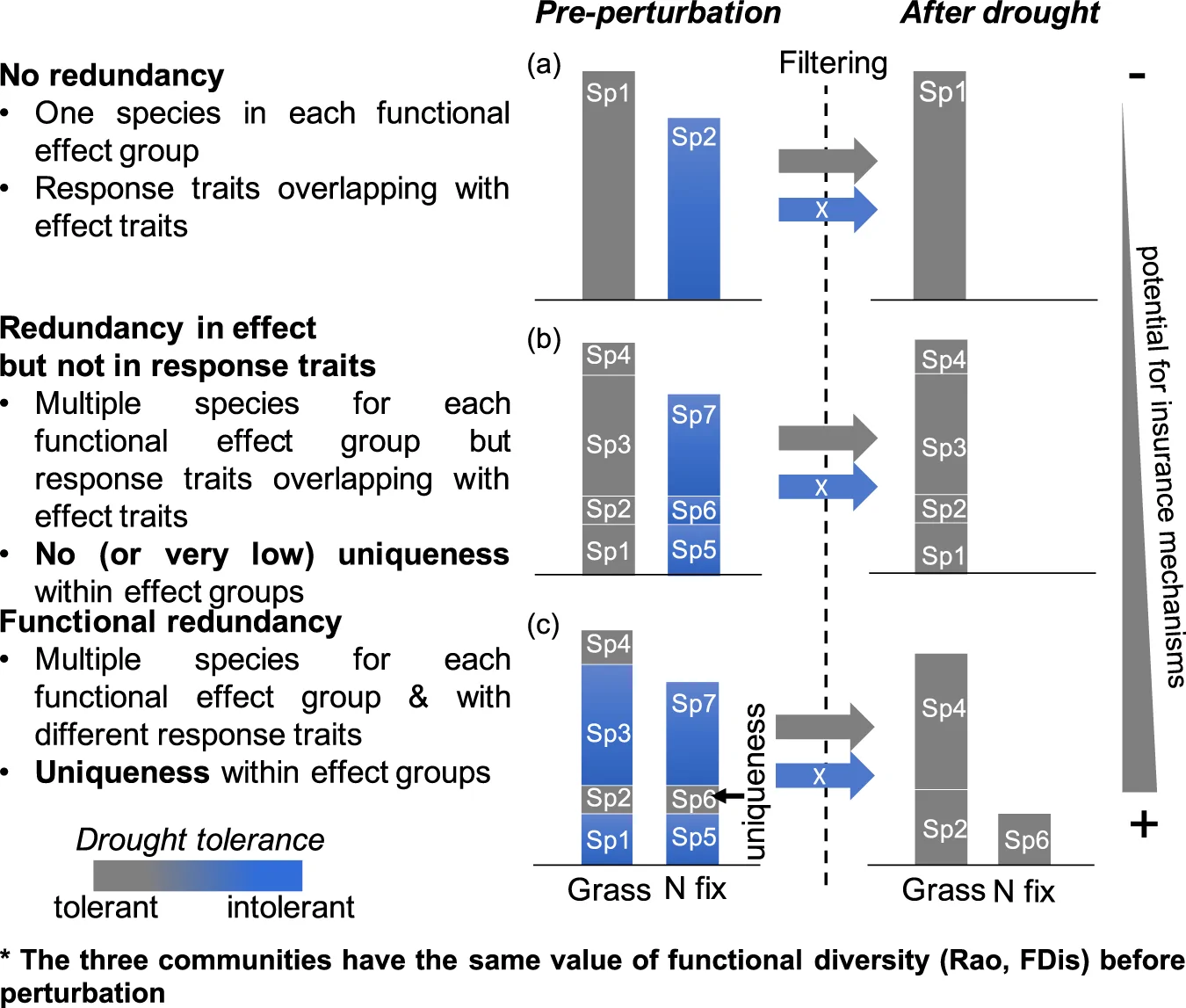
Scheme representing the role of functional redundancy on insuring ecosystem functioning after a perturbation. Here hypothetical abundances of plant species (Sp1-7) within three plant communities (**a**–**c**) are represented before and after a drought. Each species has a specific environmental requirement (represented by the level of drought tolerance) and will respond to the filter (drought) accordingly. Species might also provide a function (represented by the ability of nitrogen fixation, “N fix”). The three communities have the same dissimilarity, expressed as functional diversity (FD), in terms of their effects on the ecosystem. A hypothetical drought disturbance occurs for all three cases, leading to the filtering of the initial community species. In the “after drought” state, only drought-tolerant species survive. In cases (**a**) and (**b**) the nitrogen-fixing function is lost because of a lack of insurance mechanism provided by functional redundancy. See text for more details.

As another point, E23^1^ mentions long-term biodiversity–ecosystem function (B-EF) experiments as proof of the limitation of redundancy and its effects. Redundancy effects are often demonstrated by non-linearities in B-EF relationships. After a certain level of species diversity, a plateau is reached at which the ecosystem function does not increase with diversity. At this point, the species are considered redundant. E23^1^ notes that this plateau might disappear over time, and this could mean an absence of redundancy. It is important to recall that such experiments consist of a gradient of sown diversity across communities, which are likely not very realistic proxies for species loss^9^ and not great examples of stable “established” natural communities^10^. The biodiversity levels should be actively maintained via weeding^11^. Also, the natural decrease of species richness with time across plots, typical in these experiments, can produce bigger effects in poorer plots, exaggerating the differences, and effects, of species diversity and functional diversity across plots. The loss of species probably also increases the risk of lower insurance in the face of stronger perturbations, generally not considered in these long-term experiments^12^.

Most importantly, the mechanisms by which the plateau disappears and biodiversity effects get stronger over time in B-EF experiments^13^ merits careful discussion. The increasing effect of biodiversity on productivity over time, for example, as demonstrated by Wagg et al.^13^ cited by E23^1^, happens through the asynchrony in species’ temporal fluctuations (where the decrease of a species with certain effects on the ecosystem is compensated by the increase of another). It should be noted that the fact that asynchrony is maximizing stability in productivity actually endorses the existence of redundancy. The effect of asynchrony on stability implies that species with similar functions (effect traits) supplant each other following environmental fluctuations because of their different response traits, which supports redundancy effects^3,14^ (Fig. 1). While we agree with E23^1^ that the spatio-temporal complexity of asynchrony should not be neglected, we also highlight that it should not be taken as a proof of the lack of redundancy, but rather as an opportunity to assess the role of redundancy on stability.

We clearly agree with E23^1^ that we still cannot count on a reliable and simple way to estimate redundancy directly. Recent years have seen a proliferation of methods advancing on the matter^5,6^. However, existing indices might not yet cover the complexity of functional redundancy^8^. The estimation of functional redundancy, therefore, clearly remains an open matter, requiring similarity in effect traits but also dissimilarity in environmental preferences (Fig. 1). As E23^1^ noted, it is often not an easy task to distinguish response and effect traits, although environmental preferences are known for many species. Sometimes functional redundancy is approached by estimating the number of species with similar effects on the ecosystem, or the degree of dissimilarity between them^15^. While useful, with this approach it is often unrealistic to define groups of species with similar effects on the ecosystem without proper testing. It is also true, as pointed by E23^1^, that the more ecosystem functions considered (multifunctionality), the lower the likelihood that the same trait maximizes all functions. At the same time, the greater correlation between ecosystem functions (which is often non-negligible), the more redundancy effects could become clear^16^. This provides a set of testable hypotheses which remain to be explored consistently and do not invalidate a priori the existence of redundancy effects, but their potential weakening in some contexts.

In this context, while understanding that the difficulty in estimating functional redundancy might cause some researchers to consider its use impractical, in our view, this calls for more effort in its assessment, rather than dismissing it. Arguably, “functional similarity” is not sufficiently precise, as communities with the same functional diversity (e.g., the amount of functional dissimilarity among individuals) can vary in their potential regarding insurance mechanisms (i.e., all communities in Fig. 1 have the same dissimilarity in effect traits prior to perturbation). Recent works have attempted to decompose species diversity, functional diversity, and functional redundancy into independent components^6^, which seems a promising way ahead.

One of the bigger concerns raised by E23^1^ is that the term “redundancy” might have a negative connotation in communication and outreach, giving the idea that part of species diversity is expendable. This argument has been discussed in the past by Ehrlich and Walker^17^ who stated that this idea comes from a misinterpretation of redundancy effects described by Walker^18^. On the contrary, endorsing redundancy can be useful, as it reflects the idea that species diversity should be maximized to increase stability^3^. Redundancy is a broadly established term in other areas, such as engineering, where structural redundancy ensures the reliability of systems^19^ and it is generally an appreciated (and necessary) feature. Redundancy does not connote, in this sense, the existence of “expendable” structures, but rather that multiple protection measures increase safety. As in other areas, in biodiversity, redundancy should be taken as a promoter of safeness in ecosystems and this notion should be reinforced for broader audiences. For example, the IPBES lists “functional redundancy” in its global assessment, highlighting the insurance it provides (https://www.ipbes.net/node/41195). Thus, it is our role as specialists to avoid misinterpretations but also enable this component of biodiversity to be fully understood.

## References

[1] Eisenhauer, N., Hines, J., Maestre, F. T. & Rillig, M. C. Reconsidering functional redundancy in biodiversity research. *NPJ Biodivers.* **2**, 4–7 (2023). 10.1038/s44185-023-00015-5 PMC11332098 39242717

[2] Loreau, M. Does functional redundancy exist? *Oikos* **104**, 606–611 (2004).

[3] Mccann, K. S. The diversity–stability debate. *Nature* **405**, 228–233 (2000). 10.1038/35012234 10821283

[4] Pillar, V. D. et al. Functional redundancy and stability in plant communities. *J. Veg. Sci.* **24**, 963–974 (2013).

[5] Laliberte, E. & Legendre, P. A distance-based framework for measuring functional diversity from multiple traits. *Ecology* **91**, 299–305 (2010). 10.1890/08-2244.1 20380219

[6] Ricotta, C. et al. The ternary diagram of functional diversity. *Methods Ecol. Evol.* **2023**, 1–7 (2023).

[7] de Bello, F., Lepš, J., Lavorel, S. & Moretti, M. Importance of species abundance for assessment of trait composition: an example based on pollinator communities. *Community Ecol.* **8**, 163–170 (2007).

[8] Galland, T., Pérez Carmona, C., Götzenberger, L., Valencia, E. & de Bello, F. Are redundancy indices redundant? An evaluation based on parameterized simulations. *Ecol. Indic.* **116**, 106488 (2020).

[9] Lisner, A., Konečná, M., Blažek, P. & Lepš, J. Community biomass is driven by dominants and their characteristics—the insight from a field biodiversity experiment with realistic species loss scenario. *J. Ecol.* **111**, 240–250 (2023).

[10] Dee, L. E. et al. Clarifying the effect of biodiversity on productivity in natural ecosystems with longitudinal data and new methods for causal inference. *Nat. Commun*. **14**, 2607 (2023). 10.1038/s41467-023-37194-5 PMC10163230 37147282

[11] Roscher, C. et al. What happens to the sown species if a biodiversity experiment is not weeded? *Basic Appl. Ecol.* **14**, 187–198 (2013).

[12] Reich, P. B. et al. Impacts of biodiversity loss escalate through time as redundancy fades. *Science* **336**, 589–592 (2012). 10.1126/science.1217909 22556253

[13] Wagg, C. et al. Biodiversity–stability relationships strengthen over time in a long-term grassland experiment. *Nat. Commun.* **13**, 1–11 (2022). 10.1038/s41467-022-35189-2 PMC9751076 36517483

[14] de Bello, F. et al. Functional trait effects on ecosystem stability: assembling the jigsaw puzzle. *Trends Ecol. Evol.* **36**, 822–836 (2021). 10.1016/j.tree.2021.05.001 34088543

[15] Laliberté, E. et al. Land-use intensification reduces functional redundancy and response diversity in plant communities. *Ecol. Lett.* **13**, 76–86 (2010). 10.1111/j.1461-0248.2009.01403.x 19917052

[16] White, B. E., Hovenden, M. J. & Barmuta, L. A. Multifunctional redundancy: impossible or undetected? *Ecol. Evol.* **13**, 1–12 (2023). 10.1002/ece3.10409 PMC10427898 37593757

[17] Ehrlich, P. & Walker, B. Rivets and redundancy. *Bioscience* **48**, 387 (1998).

[18] Walker, B. H. Biodiversity and ecological redundancy. *Conserv. Biol.* **6**, 18–23 (1992).

[19] Naeem, S. Species redundancy and ecosystem reliability. *Conserv. Biol.* **12**, 39–45 (1998).

